# Does a new spatial design in psychiatric inpatient care influence patients’ and staff’s perception of their care/working environment? A study protocol of a pilot study using a single-system experimental design

**DOI:** 10.1186/s40814-018-0383-4

**Published:** 2018-12-26

**Authors:** Britt-Marie Lindgren, Jenny Molin, Mats Lundström, Maria Strömbäck, Ellinor Salander Renberg, Anders Ringnér

**Affiliations:** 1 Department of Nursing, The Caring Science Building, Umeå University, 901 87 Umeå, SE Sweden; 2 Division of Psychiatry, Department of Clinical Sciences, Umeå University, Umeå, Sweden; 3 Department of Community Medicine and Rehabilitation, Umeå University, Umeå, Sweden

**Keywords:** Activity, Environment, Feasibility, Intervention, Nursing, Process evaluation, Protocol, Single-system design, Psychiatric inpatient care, Quality interactions

## Abstract

**Background:**

Research shows that worn-out physical environments are obstacles to psychiatric inpatient care. Patients want better relationships with staff and things to do; staff want an environment that offers hope, a calm atmosphere, and joint activities. A county council in northern Sweden and Philips Healthcare partnered to create solutions to the environmental challenges of psychiatric inpatient care. One ward at a county psychiatric clinic was selected for a pilot project to test solutions that could improve the care environment for patients, staff, and relatives. The aim of the overall project is to evaluate the effects of a newly designed psychiatric inpatient ward on patients and staff in terms of quality of care and stress. In this study, we focus on the feasibility through testing questionnaires and exploring barriers to recruiting staff and patients.

**Methods:**

This study had a single-system experimental design, comparing a psychiatric unit pre- and post-implementation of the novel spatial design, using repeated measures with the same questionnaires twice a week during baseline and intervention phases. Primary outcomes were quality interactions (patients) and perceived stress (staff). Secondary outcomes were levels of anxiety and depression (patients), and stress of conscience (staff). A process evaluation was aimed to describe contextual factors and participant experiences of the new design. Data was collected using questionnaires and semi-structured individual interviews with patients and focus group discussions with staff. Both visual and statistical methods were used to analyse the quantitative data and content analysis for the qualitative data.

**Discussion:**

The findings will contribute insights into whether and how a new spatial design might contribute to quality interactions and reduced stress. This is relevant both nationally and internationally, as similar interventions are needed but sparse. The findings will be disseminated through peer-reviewed publications and conference presentations.

**Trial registration:**

ClinicalTrials.gov, NCT03140618, registered 4 May 2017

## Background

In 2015, 105,000 admissions to psychiatric inpatient care were recorded in Sweden, more than in previous years. It was also reported that patients in psychiatric inpatient care experienced poorer treatment and participated less in their healthcare than patients in somatic care [1]. Research shows that a psychiatric inpatient care ward should offer a positive atmosphere supported by an environment characterised by structure and flexibility [2–4]. Other important issues are peace, security, safety [5–9], and access to personal spaces [5, 6, 8]. Borge and Fagermoen [9] showed that an appealing, comfortable, and pleasant physical environment that felt safe and warm helped patients feel more at ease and increased their sense of self-worth and will to live, prerequisites to feeling better. Noble and Rowland [10] also reported that feeling comfortable in the environment can improve patients’ confidence, and ability to express themselves, and discovery of personal interests and opportunities.

Lindgren et al. [11] showed that patients described the environment in psychiatric inpatient care as confusing, with inconsistent procedures and regulations. There was limited space to relax and patients could not protect themselves from hearing and observing unpleasant events. Similarly, Molin et al. [12] found that patients in psychiatric inpatient care reported feeling their environment to be more stigmatising than protective. The wards were described as worn out, poor, and dirty. This made the patients feel less valued than patients treated in other specialties. Negative perceptions of the environment have also been reported by staff in the same context. In another study by Molin et al. [13], staff described that they had to represent an environment that they could not defend. They described a poor and worn out environment that in their experience had a negative impact on patients’ mental health. They wanted to offer an environment that gave hope and made them proud of their work, but instead, they felt that psychiatric inpatient care had a lower priority than other healthcare specialties.

It can be concluded that a worn-out physical environment constitutes an obstacle to psychiatric inpatient care, and that the aesthetic dimension of healthcare should be highlighted. In these situations, patients and staff share negative perceptions of the environment [12–15], leading to counterproductive care and experiences of stigmatisation among patients [12, 16]. Patients want more human relationships with staff and interesting things to do, while staff want an environment that inspires hope, creates a calm atmosphere, and offers opportunities for joint activities [17, 18].

### The light and environmental project

In 2014, a county council in northern Sweden and Philips Healthcare formed a partnership to create new innovative solutions to the environmental challenges in psychiatric inpatient care. One ward at a county psychiatric clinic was selected as a pilot project to test solutions that could improve the care environment for patients, staff, and relatives. The project was performed in several phases, beginning with an analysis of the existing facilities using a field survey and available health and environmental data. Based on the findings, a new spatial design was developed including healing lights, a sensory room, and space for physical activity. Construction of the new ward began in spring 2016 and was completed in the autumn of 2017.

Healing lights have been shown to have beneficial effects for both patients and staff. A system of lighting that automatically follows the circadian rhythm and includes multicolour elements controlled by the patients can enhance their sleep duration, mood, and general satisfaction [19]. In recent years, interest in sensory rooms has increased in many different healthcare environments, such as dementia care, brain injury rehabilitation, habilitation for people with learning disabilities, and psychiatric inpatient care [7, 20–22]. Sensory rooms are used in psychiatric inpatient care to offer patients an environment that stimulates the senses without being too demanding [20] and a room for relaxation, stress reduction, and the opportunity to develop self-soothing skills [21]. The rooms include elements such as light, paintings, photographs, coloured walls, aromatic oils, music, movies, ball quilts, textiles, and comfortable furniture [7, 20, 21, 23, 24].

The physical activity room is a social area for movement that also contains a selection of exercise equipment. Physical activity and exercise are positively related to mental health and well-being [25, 26], and research shows that such activity can help to increase self-esteem [27, 28], decrease anxiety [29], provide structure to a person’s day or week, contribute to a sense of purpose and meaning in daily life [30], and provide opportunities for social experience, commitment, and interaction [31].

### Study aim

The aim of the overall project is to evaluate the effects on quality of care and stress in patients and staff resulting from a new spatial design for a psychiatric inpatient ward. In this study, we focus on the feasibility of the project, and the objectives are to test the questionnaires and explore barriers to the recruitment of staff and patients.

#### Feasibility objectives

The feasibility objectives are to discern the following:

- The properties of the questionnaires regarding detection of change;
- Whether the chosen questionnaires are suitable for frequent measurements; and
- The plausible recruitment rates for this study.

#### Research questions

The following questions will be answered:

- Does the new spatial design influence the quality of interactions between staff and patients?
- Does the new spatial design influence patients’ mental health?
- Does access to a space for physical activity influence patients’ levels of physical activity?
- Does access to a sensory room influence patients’ mental health?
- Does a new spatial design influence staff levels of perceived stress and stress of conscience?
- Does a new spatial design influence the general activity level among patients and staff?
- Does a new spatial design influence the prevalence of coercive measures, use of PRN (as needed) medication, mean length of hospital stay for patients, and sick leave among staff?
- How do patients and staff describe their perception of the new spatial design, and how do contextual factors influence the possible changes?

## Methods

This is an intervention project using a single-system experimental design (SSED) with baseline and intervention phases [32]. SSED studies focus primarily on changes in one system (in this case the ward), and the system works as its own control. As single-system designs aim to detect changes within each system rather than to compare systems, sample sizes in this tradition are relatively small and no sample size calculations are made [33]. Data for the system will consist of aggregated measurements from patients and staff respectively on the ward [32, 33]. This means that the total number of individuals included in the study depends on (a) the number of patients admitted to the ward at each point of measurement and (b) the number of staff members working on the ward. In parallel, a process evaluation is performed to describe patient experiences and important contextual factors.

### Procedure

In the overall project, the evaluation will consist of two phases (A and B), and a follow-up, in line with the SSED. Baseline measures will be established during phase A, through outcomes measured twice a week for approximately 5 weeks. During these 5 weeks, staff and patients will not be introduced to the new environment. In phase B, staff and patients will be introduced to the new environment, and outcomes will be measured once a week over a period of 2 months.

The semi-structured focus group interviews with staff will be conducted at the end of phase B while patients will be interviewed individually very near to their discharge from the ward during phase B. Follow-up studies are planned for 6 and 12 months after phase B through interviews investigating whether the possible effects of changing the healthcare environment are sustainable [33].

### Setting

The ward at which the study is conducted initially has a total of 13 beds, with the possibility of adding 5 more; when rebuilt, there will be no possibility of adding extra beds. It is located in a building that was constructed in the late 1970s. Patients admitted, voluntarily or involuntarily, to the ward are 18 to 59 years of age and suffer from various types of mental ill-health that require specialist psychiatric treatment. The staff consists of registered nurses (RNs), mental health nurses (MHNs), and enrolled nurses. A ward manager, physicians, and a consultant psychiatrist also work on the ward, while other professions work as consultants to the ward. The ward door is locked. Certain rules and routines are followed in consideration of fixed times for medication, physicians’ rounds and other meetings, meals, smoking breaks, and walks outdoors. Medical treatment, i.e. medication, is the norm, while structured common activities, pre-planned dialogues, and other nursing interventions are sparse.

During the renovation period, the ward is relocated to an adjacent ward with the same structure as the original. Phase A, the first baseline measurements, takes place on this ward. Phase B is conducted on the rebuilt ward with the new spatial design.

### Participants

During phase A, phase B, and the follow-up, all patients admitted to the ward will be informed about the project by a research assistant. Furthermore, they will be invited to participate in the evaluation, which comprises completing questionnaires, using an activity monitor, and taking part in semi-structured interviews.

*Inclusion criteria for patients*: 18 years or older, admitted to the wards during phase A, phase B, and/or follow-up.

*Exclusion criteria for patients*: not fluent enough in Swedish to complete questionnaires and participate in interviews.

The research assistant will invite all staff working on the ward during phase A, phase B, and follow-up to participate in the evaluation.

*Inclusion criteria for staff*: regularly employed at the ward during phase A, phase B, and/or follow-up.

### Data collection

Data will be collected through questionnaires, activity measurements, ward registers, and semi-structured interviews.

#### Questionnaires

During both phases, measurements will be taken twice a week, with an assigned staff member distributing the questionnaires to the participants. Based on results from previous research [12, 34] showing that experiences of interactions with staff are related to experiences of the physical environment, the primary outcome measure for patients will be the perceived quality of their interaction with the staff. Secondary outcome measures will be levels of depressive symptoms, anxiety, satisfaction, and quality of care.

Based on results from previous research [13, 35, 36] showing that staff in psychiatric inpatient care experience stress related to inadequate physical environments, the primary outcome measure for staff will be perceived stress. Secondary outcome measures will be stress of conscience and quality of care. Demographic data will be collected for all participants.

#### Patient-related questionnaires

The Caring Professional Scale (CPS) [37] will be used to measure the quality of patient–staff interactions. The CPS consists of 15 items answered on a 5-point Likert scale. Validity and reliability has been reported as satisfactory (Cronbach’s alpha nurses 0.97) [37].

The ultra-brief self-assessment scale, the Outcome Rating Scale (ORS) [38] will be used to measure satisfaction in three areas of client functioning, specifically individual, relational, and social functioning. The specific items measured on the ORS were adapted from the OQ-45.2 [39, 40]. The three areas are each assessed on a visual analogue scale, where the respondent places a hash mark on the corresponding 10 cm line, with low estimates to the left and high to the right. The ORS has adequate validity, solid reliability, and high feasibility [38].

The self-assessment scale the Hospital Anxiety and Depression Scale (HAD) [41] will be used to measure anxiety and depressive symptoms. All items are scored on a 4-point scale. The HAD appears to be a reliable and valid method for measuring emotional distress and has been shown to be sensitive to changes in response to psychosocial interventions [41, 42]. The Swedish version was tested and showed satisfactory validity and reliability (Cronbach’s alpha 0.90) [43].

The Quality in Psychiatric Care–Inpatient (QPC-IP) [44, 45] questionnaire will be used to measure quality of care. This instrument is part of the QPC family of instruments that originate from the QPC and contains 30 items that include important aspects of patients’ perceptions of quality of care. The QPC-IP is psychometrically adequate [45] and thus recommended for evaluating patients’ experiences of the quality of psychiatric care. The instrument measures six dimensions: encounter (8 items), participation (8 items), discharge (4 items), support (4 items), secluded environment (3 items), and secure environment (3 items), on a 4-point Likert-type scale, ranging from 1 (totally disagree) to 4 (totally agree), with the option of answering ‘not applicable’ on all items. The instrument also contains several background questions about demography and general clinical characteristics, and an open question inviting any other views on quality of care [44, 45].

#### Staff-related questionnaires

The Perceived Stress Scale (PSS) [46] will be used to measure stress among staff. The PSS consists of 10 items answered on a 5-point Likert scale. The Swedish version of the 10-item PSS has proved to have satisfactory validity and reliability (Cronbach’s alpha 0.84) [47].

The Stress of Conscience Questionnaire (SCQ) [48] will be used to measure the frequencies of stressful situations and the degree to which these lead to stress of conscience among staff. The SCQ consists of nine items with two parts each. The first part uses a 6-point scale ranging from never (0) to every day (5); in the second part, a 100 mm visual analogue scale ranging from ‘No, not at all’ (0) to ‘Yes, it gives me a very troubled conscience’ (5) is used for each of the nine items. Previous studies have reported satisfactory validity and reliability (Cronbach’s alpha 0.83 for the total SCQ) [49].

The Quality in Psychiatric Care–Inpatient Staff (QPC-IPS) [44, 45] questionnaire will be used to measure quality of care. This instrument is part of the QPC family of instruments that originate from the QPC [44, 45]. Psychometric tests have been performed internationally and are ongoing nationally. Results have yet to be published.

#### Activity measurement

Staff and patients will be invited to measure their free-living physical activity during their time spent on the ward during both phase A and phase B. ActivPAL (PAL Technologies, Glasgow in Scotland, UK) is a single-site instrument validated to quantify postural allocation during sedentary, upright, and ambulatory activities. The small, lightweight device is worn on the subject’s thigh for up to 1 week at a time. The device measures sedentary time, standing and stepping activities, and the intensity of a subject’s activities. The data is captured and analysed with custom designed software.

#### Data from ward registers

Data on the use of coercion and violent interactions, PRN medication, and length of hospital stays will be collected from the ward’s existing registers.

##### Semi-structured interviews

Both patients and staff will be interviewed by the researchers in semi-structured interviews [50]. Patients will be interviewed individually while staff will participate in focus groups. During phase B, patients will be interviewed very near to their discharge from the ward, while the focus group interviews with staff will be conducted at the end of phase B. The participants will be asked to share their experiences of the ward environment and its impact on the quality of their interactions. Staff will also be asked about the ward environment’s impact on their daily work and perceived stress. Table 1 shows a flowsheet of the data collection.

**Table 1:** Modified SPIRIT-figure showing intervention and data collection flowsheet

Intervention phase	A					B									
Measurement no.	1	2	3	4	5	6	7	8	9	10	11	12	13	14	15
Assessments, patients															
CPS	●	●	●	●	●	●	●	●	●	●	●	●	●	●	●
ORS	●	●	●	●	●	●	●	●	●	●	●	●	●	●	●
HAD	●	●	●	●	●	●	●	●	●	●	●	●	●	●	●
QPC-IP	●														●
Interviews												●	●	●	●
Activity monitor		●	●							●	●				
Assessments, staff															
PSS	●	●	●	●	●	●	●	●	●	●	●	●	●	●	●
SCQ	●	●	●	●	●	●	●	●	●	●	●	●	●	●	●
QPC-IPS	●														●
Interviews												●	●	●	●
Activity monitor		●	●							●	●				
Observations at ward										●	●	●	●	●	●

*●*Measurement performed, *CPS* Caring Professional Scale, *ORS* Outcome Rating Scale, *HAD* Hospital Anxiety and Depression Scale, *QPC-IP* Quality in Psychiatric Care–Inpatient, *PSS* Perceived Stress Scale, *SCQ* Stress of Conscience Scale, *QPC-IPS* Quality in Psychiatric Care–Inpatient Staff, Supplementary data from ward registers will be collected continuously

### Sample size

Sample size for the quantitative part is estimated at 25 staff members and 30 patients. For the qualitative part, sample size is estimated at 20 staff members and 20 patients who will be purposively selected by the researchers.

### Analysis

#### Quantitative data

Demographic data for participants will be presented as means and proportions. In the SSED research tradition, focus is on changes within subjects, not between groups [33]. Thus, traditional inferential statistics are rarely used. Visual and numerical methods will be used to analyse data from the SSED study [51]. Visual inspection of the data requires comparing levels, trends, and changes between intervention phases [33]. Also, the percentage of non-overlapping data statistics will be calculated, i.e. the percentage of phase B data that overlaps with the most extreme data point in phase A [33]. In this study, data from patients and staff will be analysed separately. Measurement data for each of the questionnaires will consist of the mean ratings from all participants, patients, and staff respectively, answering at each point in time.

#### Qualitative data

Semi-structured interviews will be analysed by means of qualitative content analysis. Content analysis is a systematic method of analysing written and verbal communication [52] and can be used to analyse a person’s or a group’s experiences, reflections, and attitudes. Qualitative content analysis is an interpretive process, focusing on subject and context, considering differences and similarities between and within parts of the texts [53].

## Discussion

Combining the SSED with a process evaluation is considered highly valuable [54]. Collecting both quantitative and qualitative data will offer opportunities to describe the introduction of the new spatial design from different viewpoints. It will not only enable us to capture a wider picture of the changes in quantitative outcomes such as interaction and perceived stress, but also to evaluate experiences as described in the qualitative data. By following the process closely, we will also be able to appraise the feasibility both of implementing the new ward layout and of the research design.

The composition of the research team provides a mix of experiences with the context and the methods used for evaluation. J.M., B.M.L., and M.L. are experienced MHNs; M.S. is a physiotherapist; and E.S.R. is a professor of social psychiatry; all have experience of psychiatric inpatient and outpatient care. A.R. is a paediatric nurse with an outsider perspective on psychiatric care but is well experienced in the methods used. All authors have experience using qualitative methods.

Although this is a small-scale project, with limitations concerning generalisations, it will give a picture of the changes that might be related to the new spatial design. This study will also inform future studies about the appropriateness of proceeding with various measurements, interview questions, and other methods of data collection.

A pragmatic approach was preserved during the design of the evaluation, as it will be performed in collaboration with the management of the clinic. This could be seen as a study limitation, as the evaluation is less standardised than usual; however, it is important to conduct a feasible evaluation that would be realistic to use as a guide for other wards aiming to implement an innovative spatial design in psychiatric inpatient care. The findings will contribute insights into whether and how a new spatial environment contributes to quality interactions and reduced stress among patients and staff on a psychiatric inpatient ward.

## Abbreviations

CPS: Caring Professional Scale
HAD: Hospital Anxiety and Depression
MHN: Mental health nurses
ORS: Outcome Rating Scale
PSS: Perceived Stress Scale
RN: Registered nurses
SCQ: Stress of Conscience Questionnaire
SSED: Single-system experimental design
